# The new Brazilian Dialysis Census

**DOI:** 10.1590/2175-8239-JBN-2022-E006en

**Published:** 2022-09-02

**Authors:** Pasqual Barretti

**Affiliations:** 1 Universidade Estadual Paulista, Faculdade de Medicina de Botucatu, Botucatu, SP, Brasil

The term Census derives from the Latin word *censere*, which means to assess or rate, and has been used to describe the collection of statistical data from the inhabitants of a city, province, state, or nation^1^. Since the early 2000s, the Brazilian Society of Nephrology (SBN) has not spared efforts to conduct the Brazilian Dialysis Census with the purpose of expanding the knowledge and gathering information about dialysis and other renal replacement therapies. The Census has been an instrumental tool in the ongoing improvement of dialysis in Brazil and discussions about renal replacement therapy in general. At the same time, it has been an important element in the development of related public policies.

The Census also reflects the commitment SBN has had with providing its members and the general public with sound information, a particularly important goal in times of rampant misinformation.

Last but not least, the information collected through the Census has been referenced by Brazilian authors in publications on the subject, further illustrating its contributions to the development of science.

The 2020 Brazilian Dialysis Census published by Nerbass et al.^2^ shares relevant information and showcases comparisons against the findings of the census surveys published in 2013, 2017, 2019, and 2021. The Census shows that the absolute number of patients on chronic dialysis and prevalence continue to grow. Peritoneal dialysis utilization is still low, while the use of long-term central venous catheters in hemodialysis has increased.

The COVID-19 pandemic contributed to the increase seen in the overall mortality of patients on dialysis in Brazil, a finding first published in the 2020 Dialysis Census.

I took the liberty of looking into the 2009 Census^3^ and found that the number of individuals on dialysis grew from 77,589 to 144,779 - an 86.5% increase consistent with the reported elevations in incidence and prevalence. A noteworthy finding is the increase seen in the gross death rate, from 17.1% to 20.3%, which may be partly explained by the growing proportion of individuals with diabetes as the baseline condition causing chronic kidney disease - from 27% to 31% - and the apparent increase in the number of patients on dialysis aged 75 and older. However, one cannot disregard, in addition to the admission of patients with more comorbid conditions, the growing utilization of short or long-term central venous catheters as a vascular access, from 12.7% to 24.7%.

Chronic kidney disease is a silent condition that progresses with mild or no symptoms, which often leads to end-stage renal disease (ESRD). Consequently, many patients with ESRD are seen for the first time by a physician when they arrive at an emergency unit. The United Renal Data System 2020^4^ showed that a third of the incident patients with ESRD was admitted after being given little or no prior care.

Individuals started on unplanned dialysis are more likely to be prescribed hemodialysis with a central venous catheter^5^, a combination associated with higher infection rates and worse outcomes in the long term^6,7^.

In this context, a marked decrease was observed in the number of patients on peritoneal dialysis, from 10.5% to 7.4%. In the last decade, several studies have reported good outcomes from the utilization of unplanned peritoneal dialysis. Studies comparing unplanned peritoneal dialysis and conventional hemodialysis have reported similar outcomes in terms of patient survival^8,9^. Therefore, a more frequent use of peritoneal dialysis as early therapy might decrease the negative impact associated with the use of central venous catheters.

Notably, the article by Nerbass et al.^2^ contributed substantially to the comprehension of the impact of COVID-19 on the incidence and outcomes of patients on dialysis and described, albeit indirectly, the huge efforts made by dialysis teams to address this daunting public health crisis. The 2020 Census is a landmark document in the quality with which it presents relevant data and a must read for all active staff and healthcare workers training to work in dialysis centers. Thus, it meets every goal set out for a census survey. We must reflect about the importance of having more dialysis centers participating in the survey so as to further strengthen a tool that is indispensable to the ongoing improvement of dialysis in Brazil.
